# A comprehensive study of calcific aortic stenosis: from rabbit to human samples

**DOI:** 10.1242/dmm.033423

**Published:** 2018-06-19

**Authors:** Laura Mourino-Alvarez, Montserrat Baldan-Martin, Tamara Sastre-Oliva, Marta Martin-Lorenzo, Aroa Sanz Maroto, Nerea Corbacho-Alonso, Raul Rincon, Tatiana Martin-Rojas, Luis Fernando Lopez-Almodovar, Gloria Alvarez-Llamas, Fernando Vivanco, Luis Rodriguez Padial, Fernando de la Cuesta, Maria Gonzalez Barderas

**Affiliations:** 1 Department of Vascular Physiopathology, Hospital Nacional de Parapléjicos, SESCAM, 45071 Toledo, Spain; 2 Department of Immunology, IIS-Fundacion Jimenez Diaz, 28040 Madrid, Spain; 3 Cardiac Surgery, Hospital Virgen de la Salud, SESCAM, 45071 Toledo, Spain; 4 Department of Cardiology, Hospital Virgen de la Salud, SESCAM, 45071 Toledo, Spain; 5 Centre for Cardiovascular Science, University of Edinburgh, Queen's Medical Research Institute, Edinburgh EH16 4TJ, UK

**Keywords:** Aortic stenosis, Cardiovascular, Proteomics, Rabbit model

## Abstract

The global incidence of calcific aortic stenosis (CAS) is increasing owing, in part, to a growing elderly population. The condition poses a great challenge to public health, because of the multiple comorbidities of these older patients. Using a rabbit model of CAS, we sought to characterize protein alterations associated with calcified valve tissue that can be ultimately measured in plasma as non-invasive biomarkers of CAS. Aortic valves from healthy and mild stenotic rabbits were analyzed by two-dimensional difference gel electrophoresis, and selected reaction monitoring was used to directly measure the differentially expressed proteins in plasma from the same rabbits to corroborate their potential as diagnostic indicators. Similar analyses were performed in plasma from human subjects, to examine the suitability of these diagnostic indicators for transfer to the clinical setting. Eight proteins were found to be differentially expressed in CAS tissue, but only three were also altered in plasma samples from rabbits and humans: transitional endoplasmic reticulum ATPase, tropomyosin α-1 chain and L-lactate dehydrogenase B chain. Results of receiver operating characteristic curves showed the discriminative power of the scores, which increased when the three proteins were analyzed as a panel. Our study shows that a molecular panel comprising three proteins related to osteoblastic differentiation could have utility as a serum CAS indicator and/or therapeutic target.

## Abstract (precis)

**Summary:** Using a rabbit model of calcific aortic stenosis, we have defined a molecular panel of three proteins related to osteoblastic differentiation. Additionally, this panel has been confirmed in human samples.

## INTRODUCTION

Aortic stenosis is defined as a narrowing of the aortic valve (AV), which results in reduced blood flow to the body and ultimately in compromised heart function (Rajamannan et al., 2011). Calcific aortic stenosis (CAS), the most common etiology of aortic stenosis in Western countries, is characterized by an inflammatory process and endothelial damage caused by mechanical stress and lipid penetration, leading to fibrosis and leaflet thickening (Dweck et al., 2012). As the disease progresses, matrix remodeling and active bone formation occurs, ultimately leading to calcification (Otto, 2006). CAS has a prolonged asymptomatic period defined as aortic sclerosis, during which time calcification of the valve begins to occur, but with no elevation of the transvalvular gradient. Nevertheless, once symptoms develop, CAS is rapidly fatal as there is no effective pharmacologic treatment (Dweck et al., 2012). Patient management includes balloon valvuloplasty, which only has transient effects, and aortic valve replacement, either surgical or using transcatheter aortic valve implantation (TAVI) (Joseph et al., 2016). Accordingly, there is a great unmet need for alternative therapies to reduce the overall burden of this disease. Efforts have been directed at controlling CAS progression and towards better understanding the molecular mechanisms of CAS to provide potential indicators at early stages of the disease.

CAS is a multifactorial disease and an important challenge in its study is the presence of comorbidities, including its increased incidence with age. Animal models have been instrumental in dissecting the pathogenesis of CAS, as they allow a perfect control of external factors. In this respect, the rabbit model of aortic stenosis has been particularly useful, because of the similarities between rabbits and humans in terms of valve histology and lipoprotein metabolism (Cimini et al., 2005; Turk and Laughlin, 2004). Moreover, the existence of osteogenic cells in pathological valves has been previously described in both humans and rabbits (Kapustin et al., 2011; New and Aikawa, 2013; Liberman et al., 2008; Drolet et al., 2008).

We have previously applied different strategies to investigate the molecular changes taking place during CAS using diverse biological samples such as plasma (Mourino-Alvarez et al., 2016a; Gil-Dones et al., 2012), the secretome (Alvarez-Llamas et al., 2013) and tissue (Mourino-Alvarez et al., 2016b; Martín-Rojas et al., 2016, 2012). In this work, we have directly analyzed AV tissue from mild stenotic rabbits using a proteomic approach to identify alterations at the molecular level, which might also be reflected in plasma.

## RESULTS

### Development of CAS in the rabbit model

**Fig. 1.**
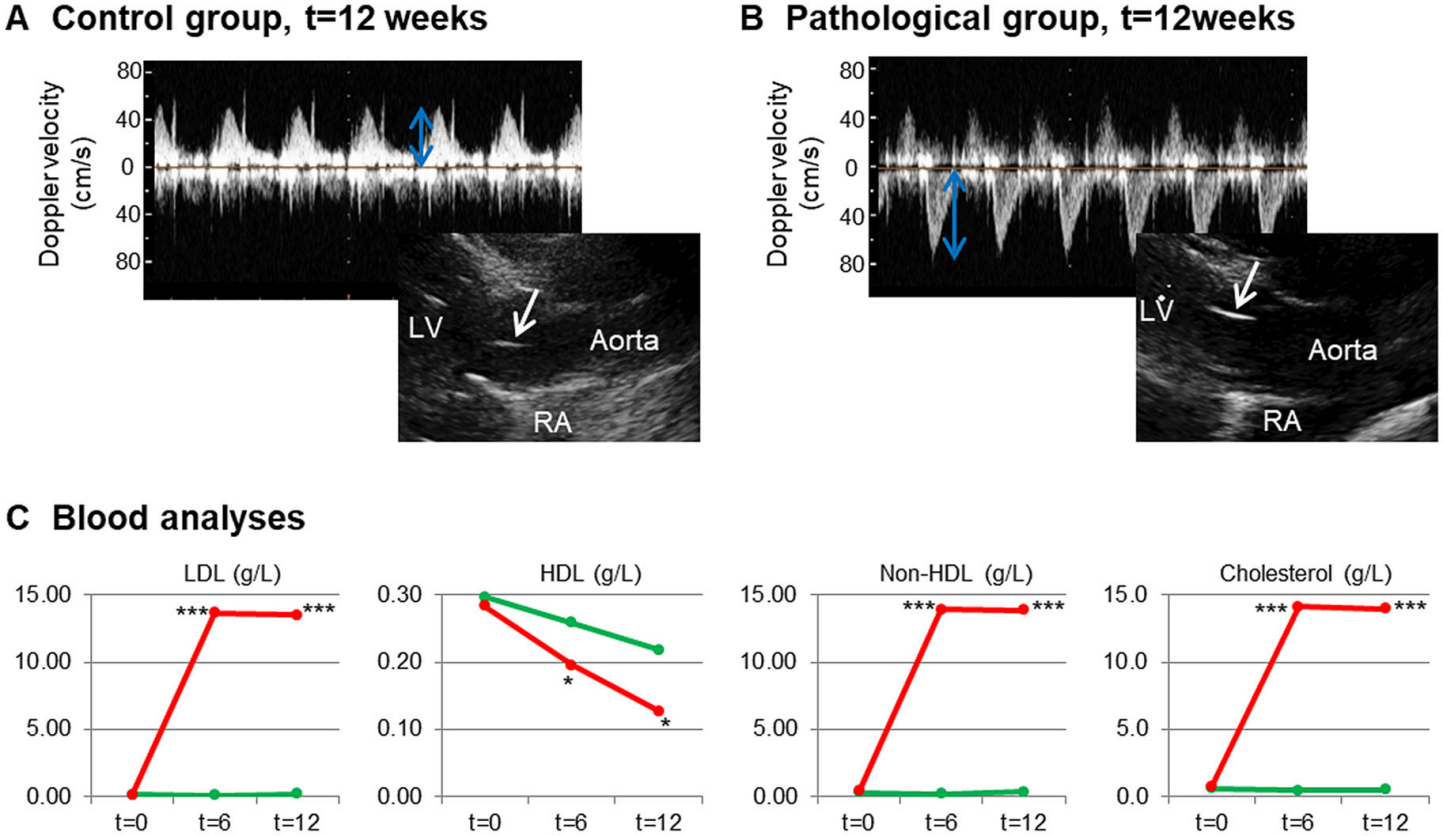
**The evaluation of CAS in the rabbit model.** (A,B) Representative echocardiograms from controls (A) and pathological rabbits (B) after 12 weeks of diet. Doppler velocity is shown in the upper images (blue arrows), whereas the aortic valves are shown by white arrows in the lower images. It can be observed that pathological rabbits have higher Doppler velocity (and, subsequently, higher transvalvular gradient) and thicker aortic valves. Aorta, left ventricle (LV) and right atrium (RA) are indicated in the figure. (C) Results from blood analyses, with significant differences marked: *P<0.05, ***P<0.001. *P*-values were calculated by comparing each corresponding time point to t=0 using a paired Student's *t*-test. Red lines, pathological group; green lines, control group.

The occurrence of CAS was evaluated by transthoracic echocardiographic examination (Table S1). As expected, after 12 weeks on a cholesterol-enriched chow plus vitamin D2 diet, all rabbits from the pathological group (*n*=7) showed higher peak gradient and thickened AVs than the control group (*n*=7; Fig. 1A,B), which confirmed the development of CAS. By contrast, the interventricular septum and left ventricular free wall, as well as left ventricular function (reflected by ejection fraction), were not significantly modified by the diet (Table S1).

Blood analyses were performed at the beginning and during the development of the study, after 6 and 12 weeks of controlled diet (Table S2 and Fig. 1C). Results showed that levels of total cholesterol were significantly higher in the pathological group than in the control group (13.99±0.29 vs 0.53±0.17 g/l; *P*<0.001). Specifically, the pathological/control ratio of low-density lipoprotein (LDL) cholesterol was >58 (13.5±0.55 vs 0.23±0.14 g/l; *P*<0.001). Significant differences were also found between pathological and control groups for high-density lipoprotein (HDL) and non-HDL cholesterol levels at the time of sacrifice (*P*<0.05).

**Fig. 2.**
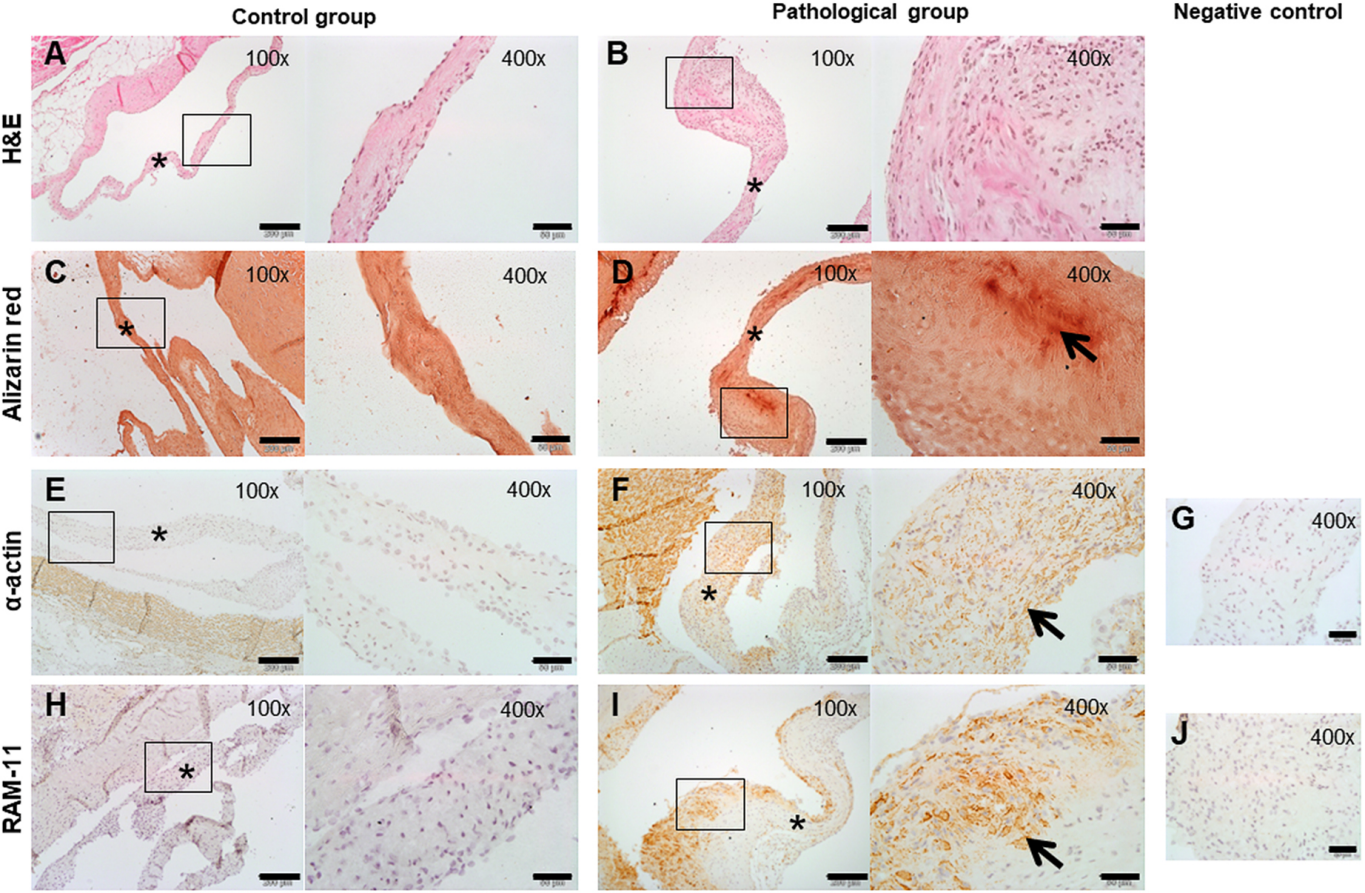
**Histology of the aortic valves from pathological and control rabbits.** (A,B) Hematoxylin and Eosin (H&E) staining reveals increased valve thickness in pathological rabbits. (C,D) Alizarin Red staining highlights the presence of calcium deposits in pathological rabbits (arrow). (E-J) Macrophage (RAM11; E-G) and SMC and/or myofibroblast (α-actin; H-J) staining is more intense in the pathological group (arrows). Scale bars: 200 µm (100× images); scale bars: 50 µm (400× images). Asterisks indicate the aortic valve.

Histological analysis of AVs from the pathological group revealed the presence of moderate calcium deposits (as shown by Alizarin Red staining), abundant infiltration of macrophages (RAM11-positive cells 2.09±1.61% in the pathological group vs 0.015±0.014% in controls; *P*=0.023) and high expression of α-actin (0.91±0.74% in the pathological group vs 0.015±0.014% in controls; *P*=0.018), which is characteristic of smooth muscle cells and myofibroblasts (Fig. 2). In addition to differences at the histological level, we noted that the pathological group had thicker AVs than the control group, as seen in the echocardiographic examination.

### Two-dimensional difference gel electrophoresis analysis and differentially expressed proteins

**Fig. 3.**
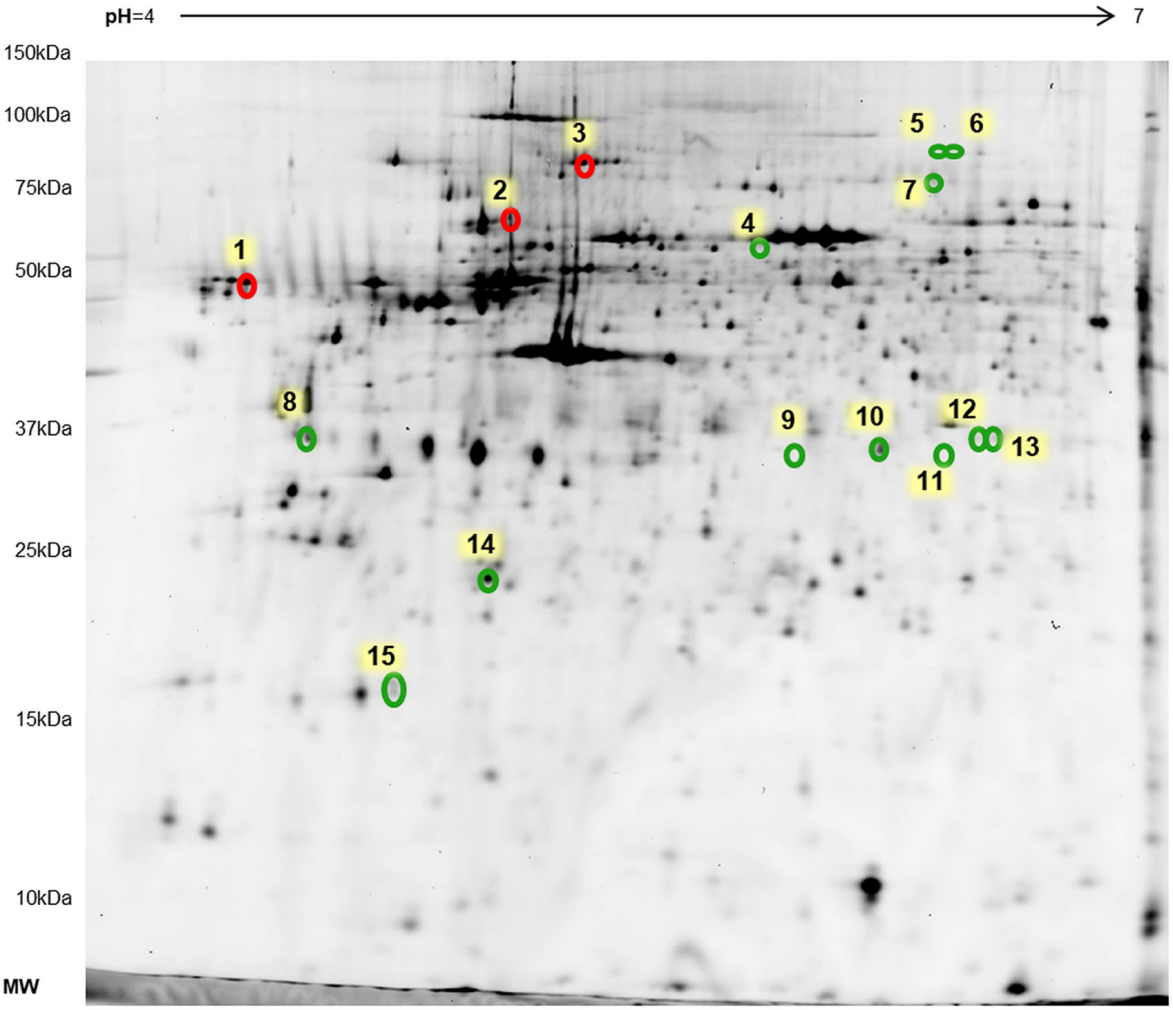
**Master gel of rabbit valve 2D-DIGE showing 15 differential protein spots between control and pathological groups.** Spots that are increased in the pathological group are shown in red, whereas spots that are decreased in this group are shown in green.

We used two-dimensional difference gel electrophoresis (2D-DIGE) in combination with tandem mass spectrometry to compare the relative abundance of proteins extracted from valve tissue in the two groups. Scanned gel images were analyzed with DeCyder Differential Analysis Software (GE Healthcare, Chicago, IL, USA), which allows detection, quantitation, matching and statistical analysis of the images. Statistical analysis (Student's *t*-test) revealed significant changes in the abundance (*P*≤0.05 and average ratio >1.5 or <−1.5) of 15 spots: five were upregulated in CAS tissue and ten were downregulated (Fig. 3). The results were analyzed using Principal Component Analysis (PCA) to reduce the complexity of the data set and to look for distinctive proteome profiles in the two study groups. As shown in Fig. S1, pathological and control valves were separated into two different groups.

Matrix-assisted laser desorption/ionization time-of-flight tandem mass spectrometry (MALDI TOF/TOF) was used to identify the significantly altered spots, which revealed eight proteins. Calreticulin, transglutaminase-2 and transitional endoplasmic reticulum ATPase (TERA) were upregulated in CAS, whereas serum albumin, tropomyosin α-1 chain (TPM-1), L-lactate dehydrogenase B chain (LDHB), myosin light chain 3 and myosin regulatory light chain 2, ventricular/cardiac muscle isoform were downregulated (Table 1).

**Table 1.**
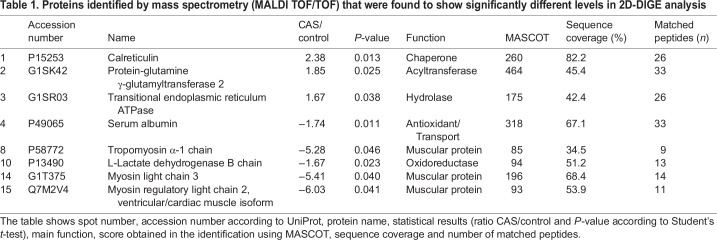
**Proteins identified by mass spectrometry (MALDI TOF/TOF) that were found to show significantly different levels in 2D-DIGE analysis**

As myosins, TPM-1 and LDHB are highly expressed in myocardial tissue (Kamakura et al., 2013; Lossie et al., 2014; Liu et al., 2016), we confirmed their presence in valve tissue by immunohistochemistry (Fig. S2).

### Selected reaction monitoring

**Fig. 4.**
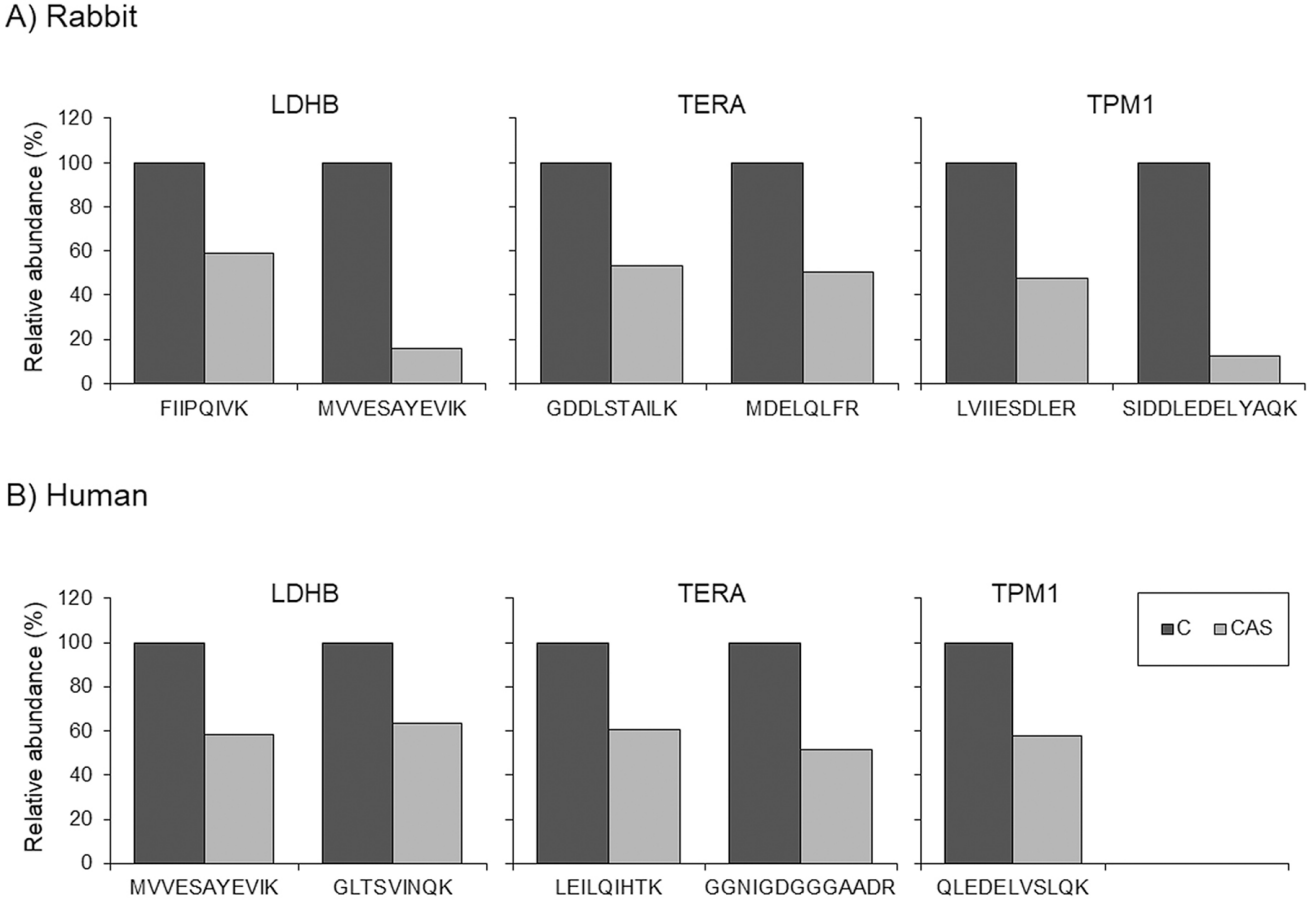
**Plasma analysis using SRM.** (A,B) Plasma analysis was performed in rabbit (A) and human (B) samples. SRM analyses allowed the measurement of tropomyosin α-1 (TMP-1), transitional endoplasmic reticulum ATPase (TERA) and L-lactate dehydrogenase B chain (LDHB). All the transitions were used to calculate the mean intensity of each peptide. Relative abundance is shown (100% corresponds to control group).

We were able to monitor five of the proteins identified in rabbit plasma by selected reaction monitoring (SRM) using liquid chromatography tandem-mass spectrometry (LC-MS/MS). Among them, three had a consistent result in the three transitions of the two peptides that were measured: TERA, TPM-1 and LDHB. TPM-1 and LDHB were found to be significantly downregulated in plasma, whereas TERA was significantly upregulated (Table 2 and Fig. 4). We also measured the levels of these proteins in plasma from patients with AV disease (*n*=34) and from control subjects (*n*=12) (characteristics of the patient study groups are shown in Table 3). This analysis showed that TERA, TPM-1 and LDHB were also significantly altered in human subjects and followed the same trend as the rabbit plasma (Table 2 and Fig. 4).

**Table 2.**
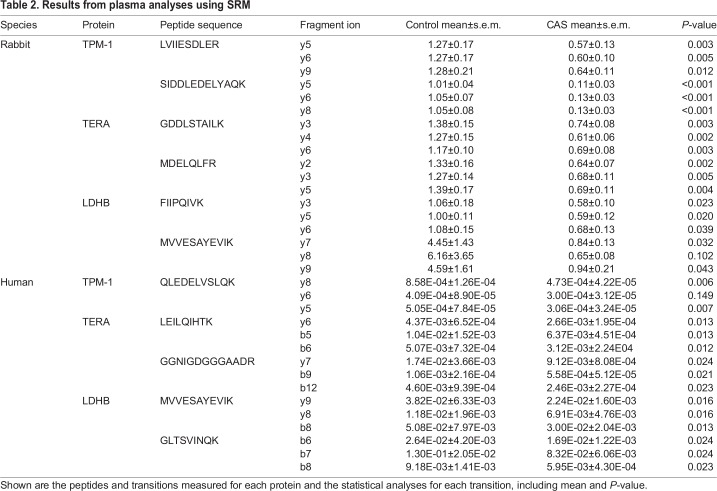
**Results from plasma analyses using SRM**

**Table 3.**
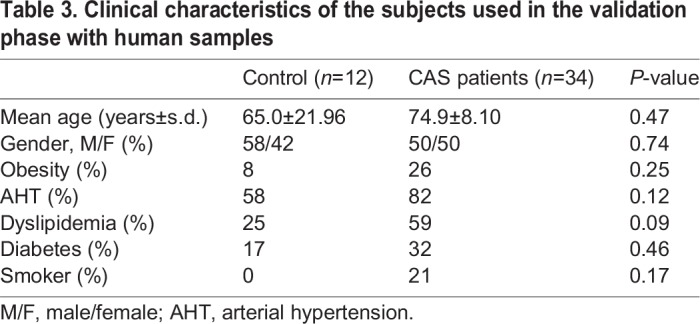
**Clinical characteristics of the subjects used in the validation phase with human samples**

**Fig. 5.**
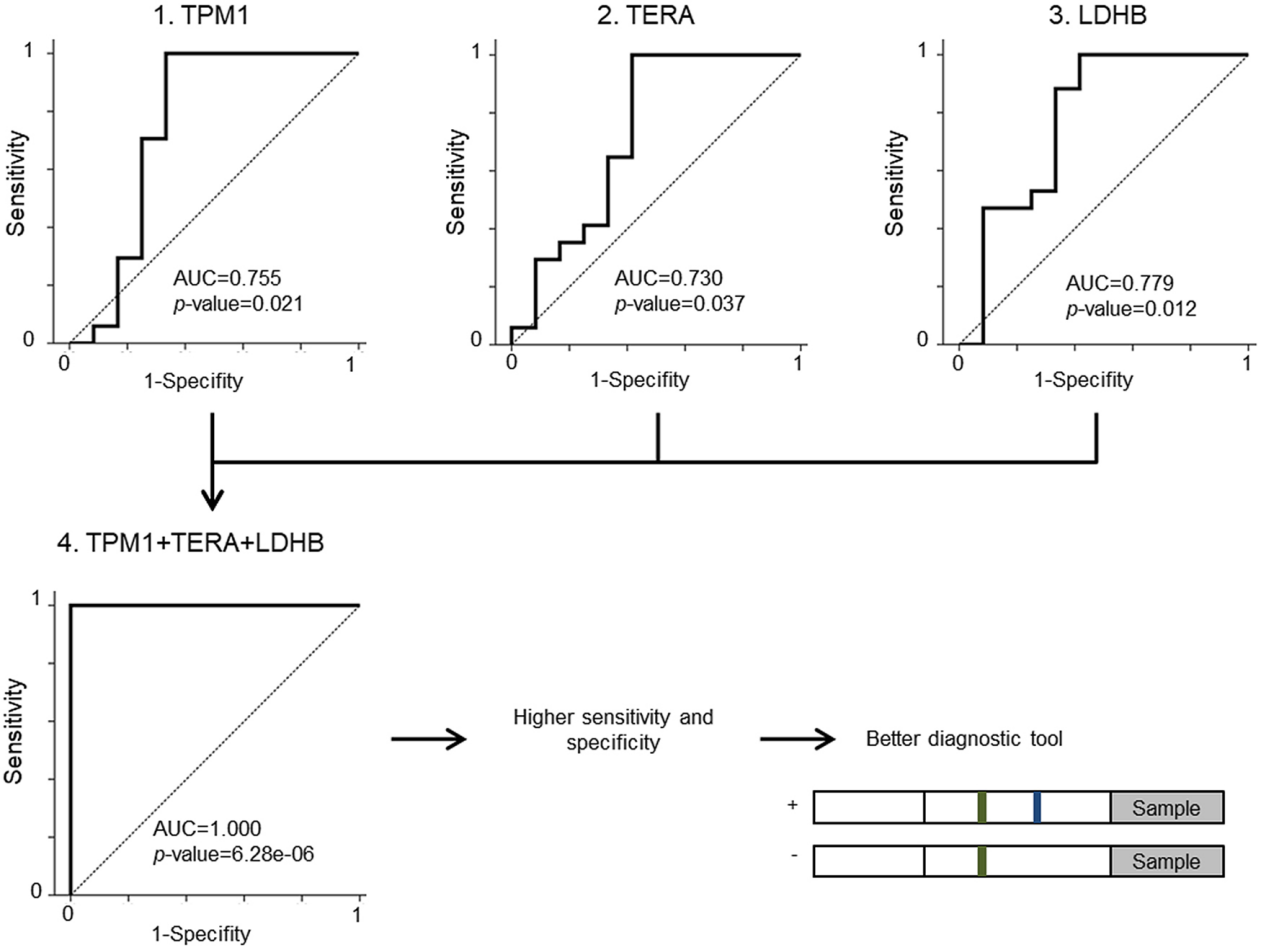
**Assessment of the sensitivity and specificity of potential markers using ROC curves.** ROC curves of tropomyosin α-1 (TMP-1), transitional endoplasmic reticulum ATPase (TERA) and L-lactate dehydrogenase B chain (LDHB) are shown in the upper part of the figure. ROC curve of the combined proteins is shown in the lower image. When these proteins are combined, the obtained panel is more sensitive and specific, thus it would be more useful than individual proteins for the development of clinical diagnostic tools. In all cases, the transition of the most significant peptide is represented. Area under the curve (AUC) and *P*-values are shown.

Results from rabbit and human plasma were used to assess the sensitivity and specificity of these potential markers by individual receiver operating characteristic (ROC) curves. In rabbits, the area under the curve (AUC) was 1.0 with significant values (*P*<0.01) for all peptides. In human plasma, the AUC was higher than 0.73 and the *P*-value below 0.037 in all cases. Moreover, when the three proteins were combined, the AUC increased to 1.0 and the *P*-value decreased to 6.28×10^−6^ (Fig. 5).

## DISCUSSION

Animal models are of great utility for studying several diseases, as they allow the control of differences between experimental groups. This is especially important in the setting of CAS given the etiology of the disease: at advanced ages, CAS is usually accompanied by other comorbidities such as hypertension or diabetes. Our rabbit model was based on a hypercholesterolemic diet supplemented with vitamin D2, which has been shown to be effective both for the study of the evolution of the early phases of AV disease (Drolet et al., 2003) and for investigating the potential prevention of progression to severe stages (Busseuil et al., 2008; Drolet et al., 2003). The increase in the transvalvular gradient confirmed the development of CAS in the pathological group, which was concomitant with a significant increase in cholesterol (total, LDL, HDL and non-HDL), characteristics that have been previously described in patients with CAS (Kamath and Rai, 2008; Akat et al., 2010). By contrast, the echocardiography study showed no development of hypertrophy or impaired systolic function of the left ventricle, which is consistent with development of mild CAS rather than severe disease (Seiler and Jenni, 1996; Mihaljevic et al., 2008). Importantly, the animal model allowed us to study valve tissue against a background of mild calcification levels. It is difficult to obtain mild calcified valves from patients, as AV replacement is recommended only at advances stages of CAS when symptoms appear owing to severe damage in valve mobility.

Our protein analysis revealed eight proteins of interest of which three were significantly altered, as shown by SRM analysis of plasma both from rabbits and patients: TPM-1 and LDHB followed the same trend in plasma and tissue, whereas TERA was upregulated in tissue and downregulated in both rabbit and human plasma.

Structural proteins such as TPM-1 are part of the cardiac muscle and the contractile cytoskeleton of various cell types, including fibroblasts and endothelial cells, and are essential for the maintenance of the endothelial barrier (Mehta and Malik, 2006). Overexpression of TPM-1 has been shown to stabilize the structure of actin filaments, helping to preserve endothelial barrier function under conditions of oxidative stress (Gagat et al., 2014). Moreover, a reduction in the expression of TPM-1 has been previously related to a decrease in the contraction of actin filaments in arteries with arteriosclerosis (Wang et al., 2011). The reduction of TPM-1 levels might also relate to the loss of AV flexibility, as occurs in arteriosclerosis, being indicative of the differentiation of the valvular interstitial cells to osteoblasts, which have no contractile ability (Lei et al., 2014; Park et al., 2009).

Valve calcification is also related to higher endoplasmic reticulum stress (ERS) (Cai et al., 2013). In a previous study, we showed that oxidized LDL promotes osteoblastic differentiation through the activation of the ERS pathway. In the present study, we found higher levels of TERA, also named valosin-containing protein, in the AV tissue of the pathological group. TERA is responsible for exporting misfolded proteins to the endoplasmic reticulum, where they accumulate and trigger ERS (Jarosch et al., 2002). In a similar way to TPM-1, the increase of TERA found in our study points to the differentiation of valvular interstitial cells to osteoblasts, which has been previously related to ERS (Cai et al., 2013).

In valve tissue, oxygen requirements exceed the amount deliverable by diffusion from the cusp surfaces alone, thus vasculature is needed to maintain a sufficient oxygen supply to the cells (Weind et al., 2000, 2002). It is therefore reasonable to assume that tissue thickening might lead to a reduction in the amount of oxygen received by these cells. In hypoxic conditions, lactate is formed from pyruvate by L-lactate dehydrogenase A chain (LDHA). LDHB is a heart-specific isoform that catalyzes the same reaction in aerobic conditions (Buono and Lang, 1999). This isoform has been shown to be downregulated in conditions of oxygen deprivation (Rossignol et al., 2003; Kay et al., 2007), which is in accordance with our results in CAS tissue and in serum from rabbits and humans with CAS.

We believe that the alterations we have found at the tissue level are significant, as they provide molecular information about the mechanisms that take place within the valve. Moreover, these proteins might serve as potential therapeutic targets to slow down the progression of the disease, although more functional analysis is needed. Of particular interest is the opposite trend we found for expression of TERA in tissue (higher in stenotic valves) and plasma (lower levels in CAS rabbits/subjects). TERA is commonly found in extracellular vesicles in several types of cells, including endothelial cells (Peterson et al., 2008) and lymphocytes (Miguet et al., 2006). TERA has also been identified in microparticles derived from human atherosclerotic plaques (Mayr et al., 2009). Given the increasing importance of extracellular vesicles in cardiovascular disease (Yin et al., 2015; Chen et al., 2018; Badimon et al., 2017), these variations in TERA levels should be further studied as they could be indicative of differential tissue and/or cell vesicle release during the development of CAS.

We have demonstrated the great potential of SRM to quantify differences in proteins across multiple samples. In rabbit samples, peptides from TPM-1 and LDHB do not have the same ratio estimations, probably because two peptides are outside the linear range of the assay (Bao et al., 2013). Special care was taken to avoid this effect in plasma samples, something crucial for the evaluation of their potential utility as diagnostic markers of these proteins. As shown in the ROC curves, each of the verified proteins has sufficient sensitivity and specificity to discriminate between control and pathological subjects (AUC>0.73), pointing to their potential utility as diagnostic markers. It should be noted that the combined measurement of the three proteins as a panel has greater discriminatory power (AUC=1.0), presenting a very high capacity to assign subjects to their corresponding study groups. Analysis in plasma samples might facilitate the translation of this panel to the clinical field, as blood samples are easy to obtain and are minimally invasive compared with biopsies and surgical procedures. Our use of LC-MS/MS for verification has not been by chance. The routine use of this technology in clinical laboratories has witnessed unprecedented growth during the past two decades owing not only to its high specificity, sensitivity and high-throughput potential, but also because it is faster and more flexible than classical immunoassays (Grebe and Singh, 2011; Leung and Fong, 2014). Therefore, it is foreseeable that LC-MS/MS will become a powerful tool in routine clinical laboratory testing.

Some limitations of this work should be highlighted. One of the most important challenges of using animal models is the notable species differences between animal models and humans. Nevertheless, CAS in vitamin D2 supplementation models is histologically and hemodynamically similar to the human disease, involving fibrosis/calcification, inflammatory response and endothelial dysfunction (Ngo et al., 2008), as well as changes in cardiac function, as shown here. Also, the rabbit model has a greater translational strength than murine models. Clearly, the analysis of human samples has the disadvantage that the underlying pathological and physiological conditions cannot be controlled, especially when studying elderly patients as they present more concomitant diseases. Finally, according to the classical development biomarker pipeline (Surinova et al., 2011), these proteins should be validated in an independent cohort of at least 100 subjects prior to clinical evaluation.

In summary, we have defined a new molecular panel that can be measured in plasma using an extremely reproducible and reliable method, such as SRM, indicating its potential for implementation in the clinic. Nevertheless, it will be necessary to perform further studies to assess some remaining aspects. It will be important to use *in vitro* and *in vivo* models to clearly define the role of these proteins in tissue. If, as we hypothesize, three of these proteins are related to osteoblastic differentiation, they might represent putative therapeutic targets to reduce calcium deposition. In addition, studies on the implications of ERS in valve calcification are scarce and it would be interesting to further our understanding of these mechanisms and to study the specific role of TERA in AV calcification. Finally, a prospective study in a larger cohort of subjects with different degrees of AV damage, from mild sclerosis to severe stenosis, should be carried out to validate the diagnostic and prognostic value of these indicators. If these candidates are suitable for clinical evaluation, it might lead to a considerable improvement in patient management, reducing the burden of CAS in society.

## MATERIALS AND METHODS

### Animal model

Male New Zealand white rabbits (*Oryctolagus cuniculus*) weighing 2–2.5 kg were divided into two groups: animals in the control group (*n*=7) were fed with normal rabbit chow; animals in the pathological group (*n*=7) were fed with 1% cholesterol-enriched chow plus 50,000 IU/kg vitamin D2 (Harlan, Indianapolis, IN, USA). All animals were fed *ad libitum* for 12 weeks (Drolet et al., 2008). Echocardiographic evaluations of the AV were performed at t=0, t=6 weeks and t=12 weeks, to ensure the establishment of CAS. Blood was drawn into EDTA tubes through the marginal vein of the ear simultaneously for the measurement of cholesterol and triglycerides. After the 12-week period, animals were sedated with an injection of ketamine (100 mg/kg) and xylazine (20 mg/kg) and then euthanized by injection of pentobarbital (50 mg/kg) directly into the heart. AVs were immediately harvested, rinsed in saline buffer and processed for analyses. When the analysis was not performed immediately, tissues were stored at −80°C.

The study was conducted in accordance with the Principles of Laboratory Animal Care and all experimental procedures were approved by the Animal Care and Use Committee of the IIS-Fundación Jiménez Díaz, according to the guidelines for ethical care of the European Community.

### Echocardiography

Ultrasound video images were obtained using the HD11XE echocardiographic system (Philips Medical Imaging, Best, The Netherlands) and a neonatal S12-4 ultrasound imaging probe, with an extended frequency range of 4–12 MHz. A parasternal long-axis view was used to measure valvular thickness and left ventricle parameters. Left ventricular ejection fraction and fractional shortening were calculated from measurements of the left ventricular internal diameter in systole and diastole. Additionally, aortic outflow velocity was registered using continuous-wave Doppler echocardiograpy from apical planes, and the peak gradient was calculated using the Bernoulli equation (Baumgartner et al., 2009).

### Tissue staining

One leaflet of each valve was fixed in 4% buffered-formalin for 24 h and then embedded in paraffin. Paraffin-embedded sections were subjected to Hematoxylin and Eosin (H&E) and Alizarin Red staining for visualization of calcium deposits. Six monoclonal antibodies were used for immunohistochemistry: RAM11 for macrophages (dilution 1:100; M0633; DAKO, Santa Clara, CA, USA), α-actin for vascular smooth muscle cells (1:100; M0851; DAKO), tropomyosin α-1 (1:25; sc-376541; Santa Cruz Biotechnology, Dallas, TX, USA), L-lactate dehydrogenase B chain (1:200; sc-100775; Santa Cruz Biotechnology), myosin light chain 3 (1:2000; ab680; Abcam, Cambridge, UK) and myosin regulatory light chain 2 (1:200; ab89594; Abcam). In control experiments, no primary antibody was added. Non-specific binding was prevented by incubation with normal goat serum (for RAM11) or 10% bovine serum albumin (for the remainder) for 1 h; non-specific peroxidase activity was blocked by incubation with 3% hydrogen peroxidase for 5 min. Incubation with primary antibodies was performed for 1 h at room temperature. The slides were then incubated with horseradish-peroxidase-conjugated polyclonal goat anti-mouse antibodies (dilution 1:100; P0447; DAKO) for 30 min, and the chromogenic reaction was developed using 3,3′-diaminobenzidine (DAB). Sections were counterstained with Hematoxylin prior to dehydration and the addition of a coverslip. For an impartial analysis of the DAB staining, an orthonormal transformation of the RGB images using an ImageJ plugin (NIH) based on Ruifrok and Johnston's method for color deconvolution was performed (Ruifrok and Johnston, 2001).

### Proteomic analysis using two-dimensional difference gel electrophoresis

One AV leaflet was ground into powder in liquid nitrogen with a mortar. Proteins were then extracted using 7 M urea, 2 M thiourea, 4% CHAPS and 1% dithiothreitol (DTT) and the homogenate was centrifuged to precipitate tissue debris. The supernatant was collected and the protein concentration determined using the Bradford assay.

Before proteomic analysis, the required amount of protein was subjected to a cleaning step by precipitation using the 2D Clean-Up kit (GE Healthcare, Chicago, IL, USA) and resuspended in rehydratation buffer (7 M urea, 2 M thiourea, 4% CHAPS, 30 mM Tris) to a final concentration of 7 mg/ml. Proteins were then labeled according to the manufacturer's instructions (GE Healthcare). Briefly, 50 µg of protein from each AV extract was labeled with 400 pmol of *N*-hydroxysuccinimide esters of Cy3 or Cy5 fluorescent cyanine dye for 30 min on ice in the dark. An internal standard containing equal amounts of all experimental samples was labeled with 400 pmol of *N*-hydroxysuccinimide Cy2 dye. Reactions were then quenched with 0.2 mM lysine. Labeled protein extracts were combined according to the experimental design (Table S3), diluted in rehydration buffer (7 M urea, 2 M thiourea, 4% CHAPS, 30 mM Tris) with 2% DTT and 1% ampholytes (IPG buffer pH 4–7, GE Healthcare) and applied to 24 cm pH 4–7 IPG strips. After passive rehydration, the first dimension was run on the IPGphor IEF II System (GE Healthcare), as described: 500 V for 1 h, a linear gradient to 1000 V over 2 h, a linear gradient to 8000 V over 3 h, and 8000 V until 96,000 V/h. After the first dimension, the strips were equilibrated in SDS equilibration buffer (1.5 M Tris-HCl pH 8.8, 6 M urea, 87% glycerol and 2% SDS) using a two-step protocol for reduction (by adding 1% DTT) and alkylation (by adding 2.5% iodoacetamide) of thiol groups. Proteins were then separated on 10% acrylamide/bisacrylamide gels using an EttanDalt Six device (GE Healthcare) (Laemmli, 1970).

### Image acquisition and analysis

Gels were scanned on a Typhoon 9400 fluorescence gel scanner (GE Healthcare) using appropriate individual excitation and emission wavelengths, filters and photomultiplier values sensitive for each of the Cy3, Cy5 and Cy2 dyes.

Images were analyzed using DeCyder software v6.5 (GE Healthcare). The Differential In-gel Analysis module co-detected the three images of each gel (spot maps from the internal standard and the two samples), measured the spot abundance in each image, and expressed these values as Cy3/Cy2 and Cy5/Cy2 ratios. The Biological Variation Analysis module enabled the matching of these spot maps, the comparison of the Cy3/Cy2 and Cy5/Cy2 ratios and the statistical analysis, to determine changes in expression levels. Only protein spots with >1.5-fold difference in abundance and with *P*-values below 0.05 (Student's *t*-test) were considered as proteins of interest. Finally, a multivariate analysis was performed by PCA using the Extended Data Analysis module. A pattern analysis hierarchical classification was also obtained using the Pearson coefficient based on the spots present in 90% of all the gels.

### In-gel digestion and protein identification by matrix-assisted laser desorption/ionization time-of-flight mass spectrometry

Differentially expressed protein spots were excised manually from the 2D-DIGE gels, which were previously stained with Oriole^TM^ fluorescent gel stain (Bio-Rad, Hercules, CA, USA). Additionally, a preparative gel using 400 µg of total protein was prepared for the identification of small or low-abundance proteins, using the same electrophoretic parameters. Spots were automatically digested with the Ettan Digester workstation (GE Healthcare) and identified at the Proteomic Unit of Hospital Nacional de Parapléjicos. The digestion was performed according to Shevchenko et al. (1996) with minor modifications and, after digestion at 37°C overnight, the peptides were extracted with 60% acetonitrile (ACN) in 0.1% formic acid. Samples were dried in a speedvac and resuspended in 98% water with 0.1% formic acid and 2% ACN. An aliquot of each digestion was mixed with an aliquot of the matrix solution (3 mg/ml matrix α-cyano-4-hydroxycinnamic acid in 30% ACN, 15% 2-propanol and 0.1% trifluoroacetic acid), and this was pipetted directly on a 384 Opti-TOF 123×81 mm stainless steel sample plate and analyzed on a 4800 Plus MALDI TOF/TOF mass spectrometer (Applied Biosystems, Foster City, CA, USA).

### MALDI-MS/MS analysis and database searching

MALDI-MS/MS data were obtained using an automated analysis loop in the MALDI TOF/TOF Analyzer. MALDI-MS and MS/MS data were combined using GPS Explorer Software Version 3.6 to search a non-redundant protein database (Swissprot 56.5) with Mascot software version 2.2 (Matrix Science, London, UK) (Perkins et al., 1999) applying the appropriate settings: 50 ppm precursor tolerance, 0.6 Da MS/MS fragment tolerance, one missed cleavage allowed, carbamidomethyl cysteines and methionine oxidation as modifications. The MALDI-MS/MS spectra and database search results were manually inspected in detail using the aforementioned software.

### Patient selection and blood extraction

Peripheral blood samples were collected from control subjects (*n*=12) and patients with severe CAS (*n*=34) who underwent scheduled AV replacement at Hospital Virgen de la Salud (Toledo, Spain). In patients, AV area (0.74±0.19 cm^2^), ejection fraction (57.13±9.83%) and mean gradient (48.16±18.06 mmHg) were assessed using transthoracic echocardiography. Blood samples were always taken before surgery. Subjects were selected to avoid significant differences between the groups in terms of six main cardiovascular risk factors: age, gender, obesity, hypertension, dyslipidemia and diabetes (Table 3). Samples from patients with bicuspid AV, concomitant aortic stenosis and aortic regurgitation or mitral valve disease were excluded.

The patient study was carried out in accordance with the recommendations of the Helsinki Declaration and was approved by the ethics committee at the Hospital Virgen de la Salud. Signed informed consent was obtained from all subjects. Blood samples (28 ml) were drawn into EDTA-containing tubes and centrifuged at 1125 ***g*** for 15 min and the resulting supernatant was immediately frozen at −80°C until analysis.

### Selected reaction monitoring

Proteins from plasma samples were reduced and alkylated by incubating with 100 mM DTT and 550 mM iodoacetamide in 50 mM ammonium bicarbonate, respectively. Proteins were digested in 50 mM ammonium bicarbonate, 15% acetonitrile with sequencing grade modified porcine trypsin, at a final concentration of 1:50 (trypsin:protein). After overnight digestion at 37°C, 2% formic acid was added and samples were cleaned with Pep-Clean spin columns (Pierce, Waltham, MA, USA). Tryptic digests were dried in a speedvac and resuspended in 2% ACN, 2% formic acid prior to MS analysis.

The LC-MS/MS system comprised a TEMPO nano LC system (Applied Biosystems) combined with a nano LC Autosampler and coupled to a modified triple quadrupole MS system (Applied Biosystems 4000 QTRAP LC-MS/MS). Three replicate injections (4 μl containing 1 μg of protein) were made for each sample using mobile phase A (2% ACN/98% water, 0.1% formic acid) with a flow rate of 10 μl/min for 5 min. Peptides were loaded onto a μ-Precolumn Cartridge (Acclaim Pep Map 100 C18, 5 μm, 100 Å; 300 μm i.d.×5 mm; LC Packings, Amsterdam, The Netherlands) to preconcentrate and desalt samples. Reverse-phase liquid chromatography was achieved on a C18 column (Onyx Monolithic C18, 150×0.1 mm i.d.; Phenomenex, Torrance, CA, USA) in a gradient of phase A and phase B (98% ACN/2% water, 0.1% FA). Peptides were eluted at a flow rate of 900 nl/min in three steps: 5–45% B for 60 min, 45–95% B for 1 min and finally 95% B for 4 min. The column was then regenerated with 5% B for 15 min. Both the TEMPO nano LC and 4000 QTRAP systems were controlled by Analyst Software v.1.5.2. The mass spectrometer was set to operate in positive ion mode with ion spray voltage of 2800 V and a nanoflow interface heater temperature of 80°C. Source gas 1 and curtain gas were both set to 20, and nitrogen was applied as both curtain and collision gases. For peptide selection, theoretical SRM transitions were designed using Skyline software v3.1.0.7382 (MacCoss Lab, Seattle, WA, USA) and Unique Peptide function from that software was used to verify that theoretical tryptic peptide sequences were proteotypic. A sample containing a mixture of all the proteins of interest was digested and analyzed with a MIDAS acquisition method, which combines a multiple reaction monitoring (MRM) scan with a full MS/MS product ion scan to allow examination of all fragment ions in the same spectrum. Peptides with six co-eluting transitions with a signal-to-noise ratio over five were considered for the analysis. Among these, the three most intense transitions for each peptide were selected for the quantification and for optimizing collision energy and dwell times to obtain maximum transmission efficiency and sensitivity for each one (Table S4). Skyline software was also used to calculate the peptide abundance on the basis of peak areas after integration.

### Statistical analysis

Statistical analyses were performed using SPSS 15.0 for windows software (SPSS Inc., Chicago, IL, USA). Continuous variables, such as age, are expressed as mean±s.d. After demonstrating normal distribution of the population using a Kolmogorov–Smirnov test, comparison of means was performed using Student's *t*-test. Differences in variables in rabbits before and after the diet were analyzed using paired Student's *t*-test. Discrete variables, such as sex or the presence/absence of risk factors, are expressed as percentages. In these cases, Fisher's exact test was used for comparison of the groups. ROC curves were generated using SPSS 15.0.

For SRM, only peptides with significant *P*-values in at least two of the three measured transitions were considered significant. Results from rabbit plasma are shown as t=12 weeks/t=0 ratio.

## Supplementary Material

Supplementary information
