# Supplementary material for: A comprehensive study of calcific aortic stenosis: from rabbit to human samples
Source: Dis Model Mech. 2018 Jun 19;11(6):dmm033423. doi: 10.1242/dmm.033423 (PMC6031362; doi:10.1242/dmm.033423)

Table SM.1.Values for echocardiographic measurements in rabbits at different times of the model (start point, 6 and 12 weeks).Ejection fraction and fractional shortening has been calculated from measurements for the left ventricular internal diameter while peak gradient has been calculated from maximal aortic outflow velocity. Changes were analyzed using paired t-test. Ao, aortic diameter; AV, thickness of the aortic valve; CAS, calcific aortic stenosis;EF, ejection fraction; FS, fractional shortening; IVSd and IVSs, thickness of the interventricular septum in diastole and systole, respectively; LVFWd and LVFWs, thickness of the left ventricular free wall in diastole and systole, respectively; PG, peak gradient.

	Group	Mean±Standard deviation			Reference interval[1]	*p*-values		
		t=0	t=6	t=12		t=0 vs t=12	t=0 vs t=6	t=6 vs t=12
**AV (mm)**	Control rabbits	0.39±0.05	0.38±0.03	0.40±0.05	-	0.98	0.63	0.42
	CAS rabbits	0.37±0.05	0.52±0.07	0.54±0.07		0.02	0.05	0.41
**IVSd (mm)**	Control rabbits	2.41±0.35	2.86±0.82	2.48±0.54	1.74-3.74	0.97	0.29	0.66
	CAS rabbits	2.22±0.18	2.68±0.81	2.93±0.68		0.06	0.11	0.91
**IVSs (mm)**	Control rabbits	4.41±0.55	4.29±0.78	3.95±0.31	2.64-5.38	0.13	0.76	0.45
	CAS rabbits	4.21±0.39	4.77±0.76	3.93±0.62		0.62	0.40	0.03
**LVFWd (mm)**	Control rabbits	3.54±3.40	2.98±0.89	2.55±0.36	1.72-3.84	0.46	0.69	0.47
	CAS rabbits	2.25±0.38	3.15±0.83	3.14±0.91		0.12	0.09	0.94
**LVFWs (mm)**	Control rabbits	3.96±0.32	4.11±0.63	3.77±0.35	2.54-4.58	0.36	0.59	0.43
	CAS rabbits	3.98±0.29	4.23±0.27	3.91±0.93		0.91	0.25	0.43
**Ao (mm)**	Control rabbits	6.85±0.34	6.89±0.76	6.92±0.73	6.39-9.41	0.90	0.90	0.77
	CAS rabbits	6.63±0.28	7.38±0.57	6.80±0.57		0.92	0.20	0.12
**EF (%)**	Control rabbits	63.75±7.00	62.62±5.65	62.47±4.83	58.73-83.51	0.91	0.72	0.74
	CAS rabbits	65.81±7.73	62.19±5.05	50.12±11.48		0.19	0.58	0.07
**FS (%)**	Control rabbits	31.79±4.81	31.31±4.07	30.77±3.34	27.39-46.95	0.88	0.83	0.60

[1] GIANNICO AT ET.AL. DETERMINATION OF NORMAL ECHOCARDIOGRAPHIC, ELECTROCARDIOGRAPHIC, AND RADIOGRAPHIC CARDIAC PARAMETERS IN THE CONSCIOUS NEW ZEALAND WHITE RABBIT.JOURNAL OF EXOTIC PET MEDICINE 24 (2015), PP 223–234

	CAS rabbits	33.40±6.12	30.89±3.25	23.47±6.74		0.20	0.59	0.07
**PG (mmHg)**	Control rabbits	1.57±0.75	1.72±0.32	1.34±0.59	0.89-4.28	0.24	0.58	0.07
	CAS rabbits	0.95±0.86	1.96±0.49	2.44±1.23		0.00	0.04	0.34

Disease Models & Mechanisms • Supplementary information

Table SM.2. Blood analysis results from rabbit model samples. Different values at the start of the model, 6 and 12 weeks are shown.Paired t-test was used to compare samples from t=0 and t=12 and corresponding *p*-values are shown.

	Control group				Pathological group			
	**t=0**	**t=6**	**t=12**	***p*-value**	**t=0**	**t=6**	**t=12**	***p*-value**
**Cholesterol (g/L)**	0.63±0.12	0.49±0.15	0.53±0.17	0.30	0.72±0.19	14.14±0.70	13.99±0.29	1.9x10-09
**Triglyceride (g/L)**	0.91±0.30	0.59±0.15	0.64±0.23	0.06	13.86±0.63	17.04±0.68	16.57±0.99	0.54
**HDL (g/L)**	0.30±0.07	0.26±0.10	0.22±0.07	0.05	0.28±0.08	0.20±0.07	0.13±0.12	0.03
**LDL (g/L)**	0.15±0.09	0.12±0.07	0.23±0.14	0.28	0.16±0.14	13.66±0.67	13.53±0.55	2.0x10-08
**Non-HDL (g/L)**	0.33±0.07	0.23±0.07	0.36±0.12	0.67	0.43±0.16	13.95±0.67	13.86±0.39	3.1x10-09

Disease Models & Mechanisms • Supplementary information

Table SM.3.Experimental design for 2D-DIGE analysis. All gels included the internal standard (IS) and two additional tissue samples from the pathological and control group. PR=Pathological rabbit; CR=Control rabbit.

Gel number	Cy2 (IS)	Cy3	Cy5
**1**	Pool	PR1	CR1
**2**	Pool	CR2	PR2
**3**	Pool	PR3	CR3
**4**	Pool	CR4	PR4
**5**	Pool	PR5	CR5
**6**	Pool	CR6	PR6

Table SM.4. List of protein monitored by SRM including the experimental parameters.

Specie	Protein Name	Accession	Peptide sequence	Precursor m/z	Product m/z	Collision energy	Fragment ion
Rabbit	TPM-1	P58772	LVIIESDLER	593.84	619.30	31.1	y5
					748.35	31.0	y6
					1073.58	31.1	y9
			SIDDLEDELYAQK	769.86	622.36	38.9	y5
					751.40	38.9	y6
					995.47	38.9	y8
	TERA	G1SR03	GDDLSTAILK	516.78	373.28	27.7	y3
					444.32	27.7	y4
					632.40	28.0	y6
			MDELQLFR	526.27	322.19	28.2	y2
					435.27	28.2	y3
					676.41	28.0	y5
	LDHB	G1TYA7	FIIPQIVK	479.31	359.27	26.1	y3
					584.38	26.0	y5
					697.46	26.0	y6
			MVVESAYEVIK	634.33	809.44	33.0	y7
					938.48	32.9	y8
					1037.55	32.9	y9
Human	TPM-1	P09493	QLEDELVSLQK	651.35	934.51	32.9	y8
					687.44	32.9	y6

Disease Models & Mechanisms • Supplementary information

Specie	Protein Name	Accession	Peptide sequence	Precursor m/z	Product m/z	Collision energy	Fragment ion
					574.36	32.9	y5
	TERA	P55072	LEILQIHTK	547.83	739.45	27.0	y6
					597.36	27.0	b5
					710.44	27.0	b6
			GGNIGDGGGAADR	558.76	603.28	27.6	y7
					685.29	27.6	b9
					942.39	27.6	b12
	LDHB	P07195	MVVESAYEVIK	634.33	1037.55	31.9	y9
					938.48	31.9	y8
					909.40	31.9	b8
			GLTSVINQK	480.28	571.34	23.1	b6
					685.39	23.1	b7
					813.45	23.1	b8

Figure SM.1. Score plot obtained in the principal component analysis, where we can appreciate a good separation of the two groups of study.

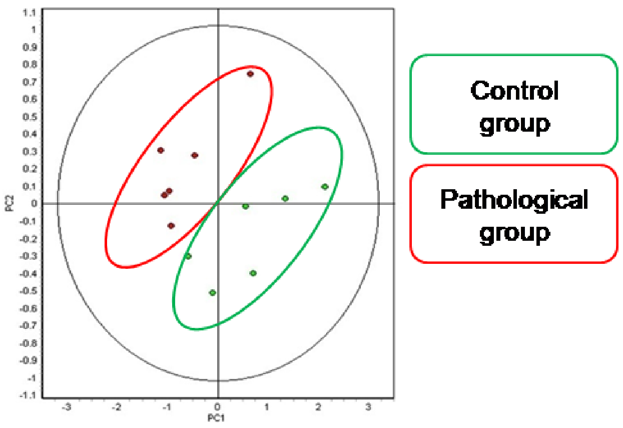

Disease Models & Mechanisms • Supplementary information

Figure 2. Histology of the aortic valves control rabbits. It is shown myosin regulatory light chain 2 (Myl2, A),myosin light chain 3 (Myl3, B), tropomyosin alpha-1 chain (TPM-1, C), L-lactate dehydrogenase B chain (LDHB, D) and calreticulin (CALR, E) staining (arrow). Scale bar corresponds to 1mm in 20x images and 100µm in 200x images. *: Aortic valve.

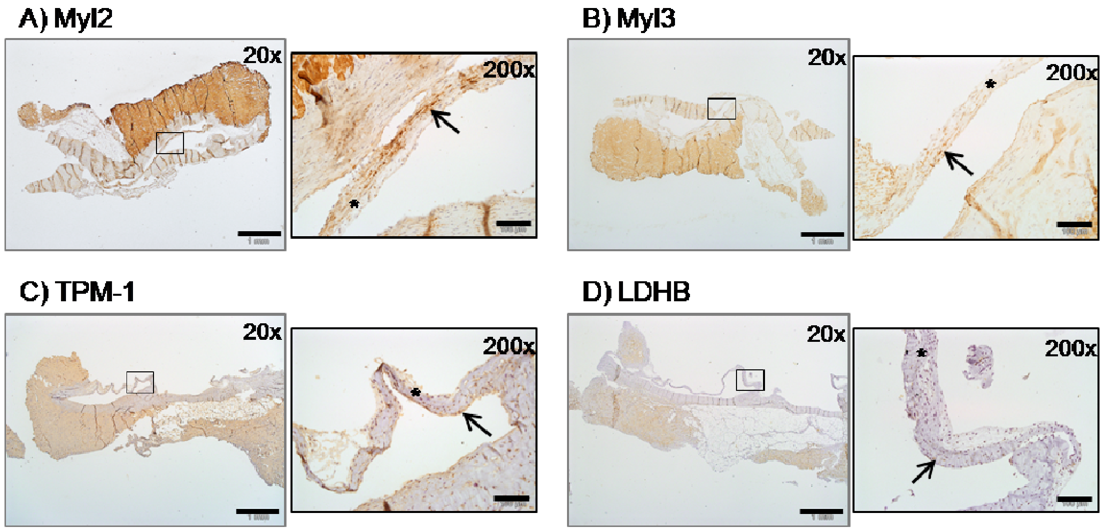

Supplement: Supplementary information [file dmm-11-033423-s1.pdf]
